# TRAP150 activates splicing in composite terminal exons

**DOI:** 10.1093/nar/gku963

**Published:** 2014-10-17

**Authors:** Kuo-Ming Lee, Woan-Yuh Tarn

**Affiliations:** Institute of Biomedical Sciences, Academia Sinica, Taipei, Taiwan

## Abstract

The spliceosomal factor TRAP150 is essential for pre-mRNA splicing *in vivo* and, when overexpressed, it enhances splicing efficiency. In this study, we found that TRAP150 interacted with the cleavage and polyadenylation specificity factor (CPSF) and co-fractionated with CPSF and RNA polymerase II. Moreover, TRAP150 preferentially associated with the U1 small ribonucleoprotein (snRNP). However, our data do not support a role for TRAP150 in alternative 5′ splice site or exon selection or in alternative polyadenylation. Because U1 snRNP participates in premature cleavage and polyadenylation (PCPA), we tested whether TRAP150 is a cofactor in the control of PCPA. Although TRAP150 depletion had no significant effect on PCPA, overexpression of TRAP150 forced activation of a cryptic 3′ splice site, yielding spliced PCPA transcripts. Mechanistic studies showed that TRAP150-activated splicing occurred in composite but not authentic terminal exons, and such an activity was enhanced by debilitation of U1 snRNP or interference with transcription elongation or termination. Together, these results indicate that TRAP150 provides an additional layer of PCPA regulation, through which it may increase the diversity of abortive RNA transcripts under conditions of compromised gene expression.

## INTRODUCTION

Eukaryotic RNA polymerase II (RNA pol II) produces mRNAs as well as a variety of non-coding RNAs. The majority of RNA pol II products necessarily undergo several interconnected processing steps to become functionally mature (1,2). A myriad of evidence indicates that nuclear processing of primary transcripts may, however, have inevitable and perhaps frequent errors that yield abortive or aberrant RNA products (3,4). Defective RNAs are generally cleared by quality-control mechanisms, but dysregulation of the quality-control system may compromise the transcriptome.

The splicing factor thyroid hormone receptor-associated protein of 150 kDa (TRAP150, also known as thyroid hormone receptor-associated protein 3, THRAP3) is an integral component of the spliceosome (5,6). Identification of TRAP150 in splicing complex B prior to the catalytic reaction (7) and in association and colocalization with the exon junction complex (8,9) suggests its role in both splicing and post-splicing. Using the splicing reporter assay, we have demonstrated that TRAP150 is essential for precursor mRNA (pre-mRNA) spicing *in vivo* and its overexpression promotes the splicing efficiency (8). We have hypothesized that TRAP150 possibly functions in a co-transcriptional manner. Moreover, it has been shown that TRAP150 represses exon skipping of CD45 by preventing polypyrimidine tract binding protein-associated splicing factor (PSF) binding to the exonic splicing silencer, suggesting its role in alternative splicing regulation (10). Most intriguingly, TRAP150, when tethered to the 3′ untranslated region of a reporter mRNA, promotes mRNA degradation in the nucleus (8). This observation implies that TRAP150 can coordinate pre-mRNA splicing and mRNA quality control (8). Nevertheless, we still have a poor understanding of TRAP150 function in gene expression. Here we report that TRAP150 interacts with cleavage/polyadenylation factors. This observation prompted us to evaluate the potential role of TRAP150 in coordinating splicing and polyadenylation.

Physical interactions between splicing factors and cleavage/polyadenylation factors may cooperatively facilitate terminal exon definition and recognition of proper cleavage/polyadenylation signals in the 3′ ends of transcripts (reviewed in Catania and Lynch; (11)). However, cryptic poly(A) sites present elsewhere in transcripts have the potential to perturb gene expression and thereby compromise transcriptome integrity. For example, polyadenylation sites that reside within introns create composite exons; activation of such sites converts an internal exon to a terminal exon, which is composed of both exon and downstream intron sequence (12). In higher eukaryotic pre-mRNAs, the size of introns ranges from hundreds to hundreds of thousands of nucleotides. Approximately 5% of introns of human genes are >200 kb in length (13). Intron size negatively correlates with gene expression efficiency (11). Large introns might be conducive to alternative splicing or aberrant processing such as premature polyadenylation, thus producing unwanted mRNA isoforms or truncated transcripts. Recent evidence indicates that the U1 small ribonucleoprotein (snRNP), in addition to its function in pre-mRNA splicing, particularly suppresses premature cleavage and polyadenylation (PCPA) of large introns (14,15). This reinforces the function of the U1 snRNP in inhibiting polyadenylation (16), and is in line with the abundance of the U1 snRNP recognition sites in the sense direction of promoter-proximal regions, which facilitates directional and productive transcription (17,18). Under conditions of transcriptional upregulation, transient shortage of U1 snRNP increases the level of PCPA transcripts (i.e. transcripts containing a composite terminal exon; (19)). Nevertheless, the detailed mechanism of this telescripting function of U1 snRNP remains unclear.

Our observation that TRAP150 associated with U1 snRNP and the cleavage and polyadenylation specificity factor (CPSF) prompted us to evaluate its function in PCPA. We found that TRAP150, although not essential for PCPA, activates cryptic 3′ splice sites in PCPA transcripts. Our finding provides a molecular mechanism for splicing following PCPA.

## MATERIALS AND METHODS

### Cell culture and transfection

HEK293 and HeLa cell culture and transfection were performed as described (8). To inhibit RNA pol II elongation, 24 h after transfection, cells were either treated with 100 μM 5,6-dichloro-1-b-d-ribofuranosyl-benzimidazole (DRB; Sigma-Aldrich Co.) or 10 μM camptothecin (Sigma) alone or with both at 37°C for 4 h. To block splicing, transfected cells were treated with 1 μM pladienolide B (PB; BioAustralis) at 37°C for 4 h.

### Plasmids

The NR3C1 minigenes including the wild-type, 5′ splice site mutant and poly(A) signal mutant were constructed into the pcDNA3.1 vector as described (14). To generate the 3′ splice site mutant 3′ss-1m, we performed PCR-based site-directed mutagenesis using the following primers: 3′ssmF: 5′-gttgctcattaacggatatcttaacg-3′; 3′ssmR: 5′-cgttaagatatccgttaatgagcaac-3′. The 3′ss-1m vector was used as the template to generate the double mutant (3′ss-2m) using the following primers: DoubleF: 5′-gtacgtaaccatttcgggttttttccttaaatagtg-3′; DoubleR: 5′-cactatttaaggaaaaaacccgaaatggttacgtac-3′. Moreover, we inserted a fragment of NR3C1 intron 2, spanning the region from 157 bp upstream to 51 bp downstream of the poly(A) signal, into the intron or the second exon of the β-globin intron-containing CAT reporter gene (20) to generate pcDNA-iCAT(In) and pcDNA-3′eCAT(In), respectively. To prevent the interference of the two nearby poly(A) signals, the bovine growth hormone poly(A) signal of the pcDNA-3′eCAT(In) was mutated (from aataaa to ggatcc) by site-directed mutagenesis. The expression vectors encoding FLAG- and HA-tagged TRAP150 and their derived mutants, and SRm160 have been previously described (8). The U1 decoy expression vectors were obtained from A. R. Krainer (Cold Spring Harbor Laboratories).

### Antibodies

Antibodies against the FLAG epitope, hRrp6 and U2AF65 were from Sigma-Aldrich, antibodies against BCLAF1 (A300-608A), CPSF73 (A301-091A), CPSF160 (A301-580A), CstF64 (A301-092A), TRAP150 (A300-956A), Xrn1 (A300-443A) and Xrn2 (A301-102A) were from Bethyl Laboratories, anti-pol II C-terminal domain (4H8) was from Abcam, anti-poly(A) polymerase (H-300) was from Santa Cruz Biotechnology, anti-HA epitope was from Bioman Scientific and anti-α-tubulin (DM-1A) was from Thermo Scientific. The antibody against the U1-70K protein was a gift of R. Luhrmann (University of Göttingen).

### Immunoprecipitation and mass spectrometry

HEK293 cells (∼3 × 10^6^) were transiently transfected with 10 μg of the FLAG-TRAP150 expression vector by calcium phosphate. At 48 h post-transfection, cells were lysed in 1 ml of hypotonic buffer (10 mM Tris-HCl, pH 7.5, 10 mM NaCl, 10 mM EDTA, 0.5% (w/v) Triton X-100) at 4°C for 15 min followed by addition of 37.5 μl of 4 M NaCl to the final concentration of 150 mM. After centrifugation at 13 400 × g for 15 min, the soluble fraction was subjected to immunoprecipitation using anti-FLAG (8). For mass spectrometry analysis of TRAP150-interacting proteins, ∼1.5 × 10^7^ FLAG-TRAP150-expressing HEK293 cells were used. Immunoprecipitated proteins were separated by SDS-polyacrylamide gel electrophoresis and stained with SYPRO Ruby (Bio-Rad). The bands of interest were excised, trypsinized and subjected to liquid chromatography coupled with tandem mass spectrometry (LTQ XL, ThermoFinnigan).

### Glycerol gradient sedimentation analysis

The 15–40% glycerol gradient was prepared in buffer containing 20 mM HEPES (pH 7.9), 0.2 mM EDTA and 150 mM KCl. HeLa cell lysate (500 μl; see above) was layered onto a 12-ml gradient and centrifuged in a Beckman SW41 rotor at 36 000 rpm at 4°C for 36 h. Gradient fractions (500 μl each) were manually collected from top to bottom, and proteins were precipitated with 10% trichloroacetic acid. To determine the phosphorylation status of TRAP150, 250 μl of fraction 21 was treated with 80 units of alkaline phosphatase (Roche) at 37°C for 30 min and precipitated with 10% trichloroacetic acid.

### RNP immunoprecipitation and northern blotting

As described above, HEK293 cells were transfected with each of the FLAG-tagged protein expression vectors followed by immunoprecipitation using anti-FLAG. Co-precipitated RNAs were recovered from beads using Trizol reagent (Life Technologies). One-fifth the amount of the RNAs was separated by a 6% denaturing polyacrylamide gel followed by northern blotting analysis using ^32^P-labeled antisense riboprobe to detect spliceosomal snRNAs (21).

### *In vivo* splicing, reverse transcription-PCR (RT-PCR), quantitative RT-PCR (qRT-PCR) and rapid amplification of cDNA 3′ ends (3′ RACE)

To evaluate the effect of TRAP150 in PCPA of endogenous genes, 10 μg of pcDNA3.1-based expression vector encoding FLAG-tagged TRAP150 or deletion mutant was transfected into ∼5 × 10^6^ of HEK293 cells. We used pCEP4-based human influenza hemagglutinin epitope (HA)-tagged TRAP150 expression vectors for the NR3C1 minigene assay, because the minigene was derived from pcDNA3.1. In brief, 0.5 μg of the NR3C1 minigene or a CAT(In)-derived splicing reporter was co-transfected with 2.5 μg of the HA-tagged TRAP150 expression vector into ∼1 × 10^6^ HeLa cells. For U1 blockage, the U1 decoy plasmid (1, 2 or 4 μg) was included. After 24 h, cells were harvested and RNAs were recovered using TRIzol reagent. Reverse transcription was performed as described (8), except that the oligo dT18-XbaKpnBam primer (14) was used for the first-strand cDNA synthesis. The cDNAs were subjected to quantitative PCR (qPCR) or 3′ RACE. For qPCR, reactions were prepared using SensiMix SYBR^®^ No-ROX kit (Biolone). PCR was performed on the LightCycler 480 (Roche) using the default run mode with SYBR Green I. *Actin* served as reference. For the 3′ RACE analysis, the first-round PCR, >35 amplification cycles were performed using the gene-specific forward primer and the reverse XbaKpnBam adaptor primer. All possible poly(A) containing transcripts of each gene could be selectively amplified. Subsequently, 10 and 15 cycles of nested PCR were performed for mRNA and PCPA, respectively, using the gene-specific chimeric reverse primers (Supplementary Table S1), each of which contained a string of 12 Ts followed by several residues complementary to the sequence immediately upstream of the putative cleavage site. To detect the cryptically spliced products of PCPA (hereafter termed CSPP) product, 20 amplification cycles of PCR were performed. The annealing temperature used for the first-round PCR reaction was 55°C whereas it was lowered to 50°C for the nested PCR, because a low GC content primer was used. For the knockdown experiments, cells were transfected with 100 nM short interfering RNA (siRNA) and incubated for 48 h. Cells were further transfected with the NR3C1 minigene and an effector expression vector and then incubated for another 24 h. Total RNA was isolated using Trizol reagent, followed by 3′ RACE analysis. All siRNAs used in this study were purchased from Life Technologies, and their sense-strand sequences were as follows: si-BCLAF1: 5′-gguuauagaccugucuggaauagaa-3′; si-CPSF73: 5′-aggugcaguacagaagguuucuaaa; si-hRrp6: 5′-ucuucuauaggucuguauaacuggg; si-Luc.: 5′-ggatttcgagtcgtcttaatgtata; si-TRAP150: 5′-ccaaaagguauaagcuccgagaugauu; si-Xrn1: 5′-gggaucuggaaagaugcaauacuuu; si-Xrn2: 5′-gagaggagcauugaugacuggguuu.

### Bioinformatic analysis

The human intronic sequences were extracted from the UCSC human genome browser (hg19; http://genome.ucsc.edu/). We searched for the intronic promoter-proximal poly(A) signals using the following criteria: poly(A) signals located (1) within 5 kb downstream of the transcription start site, (2) in the first or second intron of at least 1 kb in length and (3) within 1 kb downstream of the 5′ splice site, and thus identified 8949 introns containing at least one promoter-proximal poly(A) signal. For the 3′ splice site analysis, we selected promoter-proximal poly(A) signals that are located <500 bp downstream of the 5′ splice site. Among the selected 4761 genes, we searched for those containing an upstream 3′ splice site consensus sequence (i.e. 7-mer branch site followed by 14-mer 3′ splice site) (22,23) using the Human Splicing Finder (24). Finally, we identified 2849 genes that contain at least one potential 3′ splice site with a consensus value of the 3′ splice site and branch site above the threshold (39.41 and 50.16, respectively).

## RESULTS

### TRAP150 interacts with pre-mRNA 3′ end processing factors

**Figure 1.**
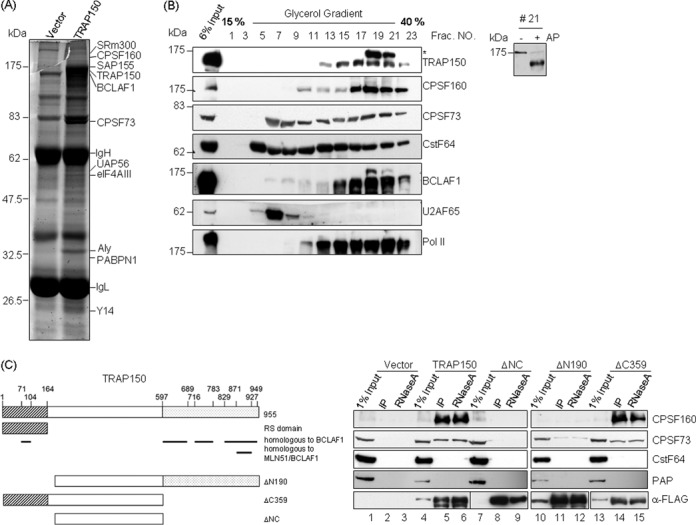
TRAP150 interacts and co-fractionates with the CPSF complex. (**A**) The empty or FLAG-TRAP150-expressing vector was transiently transfected into HEK293 cells. The anti-FLAG co-precipitated proteins were stained with SYPRO Ruby. Proteins identified by mass spectrometry are indicated; IgH and IgL represent immunoglobulin heavy and light chains, respectively. (**B**) HeLa cell lysates were fractionated by 15–40% glycerol gradient sedimentation. Fractions were manually collected from top to bottom, followed by immunoblotting using antibodies specific to the indicated RNA processing factors (left panel). Right panel shows TRAP150 in mock-treated or alkaline phosphatase (AP)-treated fraction 21. (**C**) The diagram depicts the domain structure of TRAP150 and its truncated forms (left). The hatched and gray regions represent the RS domain and the homology domain, respectively. The thick lines indicate the BCLAF1 and MLN51 homology regions. The right panel shows the result of immunoprecipitation. Full-length or truncated FLAG-TRAP150 was transiently expressed in HEK293 cells. Immunoprecipitation was performed using anti-FLAG; the precipitates were mock- (IP) or RNase A-treated followed by immunoblotting with the indicated antibodies. PAP, poly(A) polymerase.

To have a better understanding of the function of TRAP150, we screened for its interacting partners. We transiently expressed FLAG-tagged TRAP150 in HEK293 cells and then prepared cell lysates for immunoprecipitation with anti-FLAG. Mass spectrometric analysis identified several splicing factors, components of the exon junction complex and, notably, several CPSF members (Figure 1A). To assess whether TRAP150 physically associates with the polyadenylation machinery, we performed glycerol gradient sedimentation. The polyadenylation factors CPSF73 and CstF64 showed a broad distribution on a 15–40% glycerol gradient and co-fractionated with U2AF65 in the lighter fractions (Figure 1B, fractions 5–9). TRAP150 distributed in the heavier fractions, in which CPSF160 in addition to CPSF73 and CstF64 were detected (Figure 1B, fractions 17–21). Furthermore, we observed that RNA pol II and BCLAF1 (BCL2-associated transcription factor 1), which shares a homologous domain (48% overall identity) with TRAP150 and functions analogously (Figure 1C; (8)), also co-fractionated with TRAP150, suggesting the existence of a supra-complex containing RNA pol II as well as factors involved in splicing and polyadenylation. Moreover, protein phosphatase treatment confirmed that a slower migrating TRAP150 band (Figure 1B, left panel, fractions 19 and 21, marked by *) represented a phosphorylated form of TRAP150 (Figure 1B, right panel).

We then examined whether TRAP150 directly interacts with the polyadenylation factors. Immunoprecipitation of FLAG-TRAP150 from the lysate of transfected HEK293 cells followed by immunoblotting revealed that a large fraction (∼10–20%) of CPSF160 co-precipitated with TRAP150 regardless of the presence of RNase (Figure 1C, lanes 5 and 6). In addition, TRAP150 also interacted with CPSF73, albeit to a lesser extent, but not with CstF64 or poly(A) polymerase (Figure 1C, lanes 5 and 6). This result may suggest a specific interaction between TRAP150 and the CPSF complex. Moreover, using truncated TRAP150 proteins as bait, we observed that the N-terminal RS repeat-containing domain of TRAP150 was important for its interaction with CPSF, whereas the C-terminal 359-residue region was dispensable (Figure 1C, lanes 10–15). Notably, the splicing activity of TRAP150 also requires the N-terminal but not the C-terminal domain (8), suggesting that TRAP150 provides a link between splicing and polyadenylation.

### TRAP150 preferentially associates with U1 snRNP

**Figure 2.**
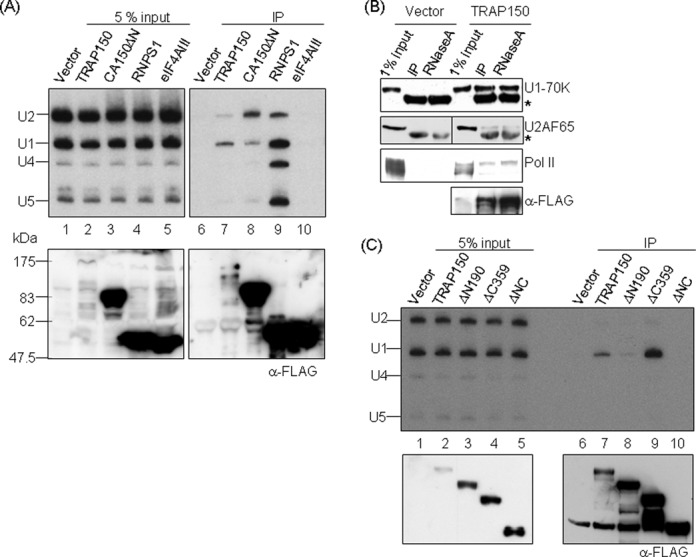
TRAP150 associates with U1 snRNP. (**A**) Expression vectors encoding different FLAG-tagged proteins as indicated were each transiently transfected into HEK293 cells. Input (5%) and anti-FLAG-immunoprecipitated RNAs (IP) were separated by acrylamide gel electrophoresis followed by northern blotting using specific snRNA probes. The immunoprecipitated proteins were detected using anti-FLAG (bottom). (**B**) Transfection and immunoprecipitation were performed as in panel A. Input and mock-treated (IP) or RNase A-treated co-precipitates were subjected to immunoblotting using specific antibodies as indicated. Asterisk represents the immunoglobulin heavy chain. (**C**) FLAG-tagged full-length and truncated TRAP150 proteins were each overexpressed in HEK293 cells and immunoprecipitated from cell lysates. Co-precipitated RNAs and proteins were subjected to northern blotting and immunoblotting as in panel A.

To test whether TRAP150 mediates the interaction between spliceosomal snRNPs and CPSF, we immunoprecipitated FLAG-TRAP150 and recovered co-precipitated RNA for northern blot analysis using probes complementary to snRNAs. The result showed that TRAP150 preferentially associated with U1 snRNA in comparison to other RNA processing factors (Figure 2A, lane 7). Immunoprecipitation revealed that FLAG-TRAP150 co-precipitated more amounts of the U1-70K protein than U2AF65 (Figure 2B), confirming a higher affinity of TRAP150 in the interaction with the U1 snRNP. We reasoned that the minimal interaction between TRAP150 and U2AF was possibly due to the association of TRAP150 with the spliceosome. Next, we determined the TRAP150 domains involved in the interaction with U1 snRNP. The result showed that the N-terminal domain was essential (Figure 2C, lane 8), which was expected. Moreover, the observation that the C-terminally truncated TRAP150 reproducibly co-precipitated a higher amount of U1 snRNP (Figure 2C, lane 9) than did full-length TRAP150 suggested that this truncated form is inefficiently released from splicing complexes.

### TRAP150 is not essential for the expression but activates the splicing of PCPA transcripts

The interactions between TRAP150 and U1 snRNP as well as components of CPSF initially prompted us to examine whether TRAP150 could modulate alternative splicing or polyadenylation. Using minigene-based splicing assays, we observed that overexpression or knockdown of TRAP150 had no significant effect on either 5′ splice site usage of the adenovirus E1a transcript or exon selection of α-tropomyosin (Supplementary Figure S1A and B). To our surprise, TRAP150 could modulate terminal exon selection of the calcitonin/calcitonin gene-related peptide (CT/CGRP) minigene (25) (Supplementary Figure S1C). Utilization of CT/CGRP exon 4 is promoted by U1 snRNP binding to the pseudoexon-like enhancer in the downstream intron (25). At present, we inferred that TRAP150 suppressed exon 4 inclusion by sequestering the U1 snRNP or preventing U1 snRNP access to the enhancer; the hypothesis warrants further investigation.

**Figure 3.**
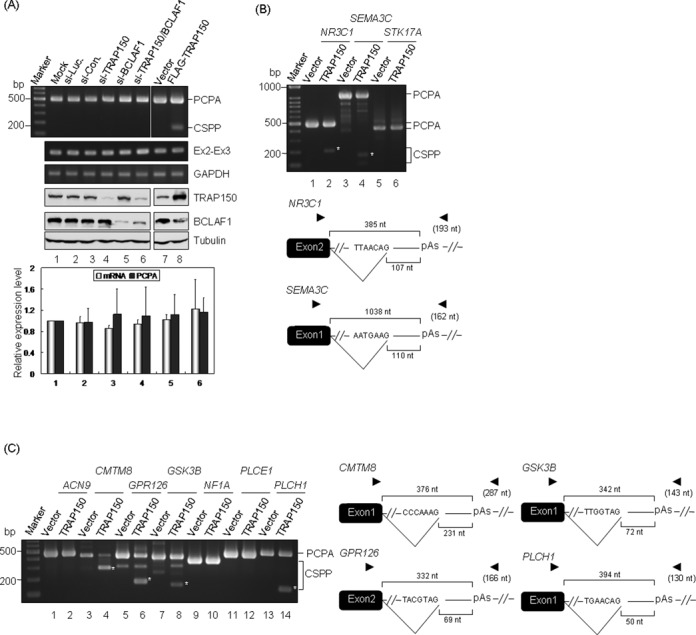
TRAP150 activates splicing of PCPA transcripts. (**A**) Lanes 1–6: HeLa cells were mock-transfected (Mock) or transfected with siRNA targeting luciferase (si-Luc.), low-GC control siRNA (si-Con.), or siRNA targeting TRAP150 (si-TRAP150), BCLAF1 (si-BCLAF1) or both (si-TRAP150/BCLAF1). Lanes 7 and 8: cells were transfected with the empty or FLAG-TRAP150-expressing vector. The PCPA transcript of *NR3C1* was detected by 3′ RACE. *NR3C1* mRNA was detected by primers targeted to exons 2 and 3 (Ex2–Ex3), and GAPDH served as the loading control. Knockdown of TRAP150 and BCLAF1 was evaluated by immunoblotting using specific antibody to each. Tubulin served as the control. The bar graph shows the relative expression level of the mRNA and PCPA transcripts (lanes 1–6, above) that were determined by qPCR; the expression level of each transcript was normalized to the mock control; the average and standard deviation were obtained from three independent experiments. (**B**) Transfection was as in lanes 7 and 8 of panel A. The PCPA and CSPP transcripts of *NR3C1*, *SEMA3C* and *STK17A* were detected by 3′ RACE using the appropriate primers (Supplementary Table S1). Diagrams show the position of the polyadenylation signals (pAs) and primary cryptic 3′ splice sites in the PCPA *cis-*element-containing intron. Primers used for CSPP transcript detection are indicated by arrowheads and the size of each amplicon is shown in parenthesis. (**C**) Analysis of the selected gene that may undergo PCPA (see Materials and Methods) was essentially performed as in panel B. A representative result is shown in panels B and C; similar results were observed in at least three independent experiments and the asterisk depicts the CSPP transcripts.

On the other hand, we evaluated alternative polyadenylation site usage of endogenous *CDC42*, *GABPB1* and *UBAP2L* mRNAs in TRAP150 siRNA transfected HEK293 cells. While ∼70% of TRAP150 was depleted, no obvious change was detected in the alternative polyadenylation isoform expression of these three genes (Supplementary Figure S2). Nevertheless, the role of U1 snRNP in PCPA (14) prompted us to assess whether TRAP150 modulates the expression of PCPA transcripts. We first examined several previously known PCPA transcripts including *NR3C1* (nuclear receptor subfamily 3, group C, member 1; (14)) in TRAP150-depleted HEK293 cells. The result showed that TRAP150 depletion had no significant effect on PCPA of the *NR3C1* gene (Figure 3A, lane 4). To evaluate the possibility that BCLAF1 may compensate for the loss of TRAP150 (8,9), we depleted BCLAF1 or both factors simultaneously. The level of the *NR3C1* PCPA transcript was not significantly changed (Figure 3A, lanes 5 and 6). Similar results were observed on other genes (Supplementary Figure S3; gene name abbreviations are listed in Supplementary Table S2). However, transient overexpression of TRAP150 in HEK293 cells generated additional products of *NR3C1* and *SEMA3C* (Figure 3A and B, lanes 1–4); this effect was not observed with BCLAF1 (Supplementary Figure S4). Cloning and sequencing confirmed that these TRAP150-induced fragments represented CSPP (Figure 3B and C). As expected, only PCPA transcripts of *STK17A* which contains no consensus 3′ splice site could be observed when TRAP150 was overexpressed (Figure 3B, lane 6). Neither knockdown nor overexpression of TRAP150 affected the level of the spliced *NR3C1* mRNA (Figure 3A, Ex2–Ex3), suggesting that CSPP may not result from alternative splicing of the *NR3C1* pre-mRNA but rather from PCPA. In fact, splicing of PCPA transcripts is not unprecedented (19), but its underlying mechanism has not been characterized.

To evaluate whether TRAP150 generally participates in the splicing of PCPA transcripts, we performed bioinformatic analysis to identify additional PCPA transcripts that might undergo splicing. We found that ∼40% of human genes contain intronic poly(A) signals that are located within a <5-kb region from the transcription start site. We then focused on those with features similar to the PCPA property of *NR3C1*, i.e. the polyadenylation site of PCPA is located <500 nt downstream of the 5′ splice site, and potential 3′ splice sites exist between these two sites. Finally, we selected seven candidates, *ACN9*, *CMTM8*, *GPR126*, *GSK3B*, *NF1A*, *PLCE1* and *PLCH1* (Supplementary Figure S3A) to examine their propensity to undergo PCPA. PCPA transcripts were detected in all seven genes, even without blocking U1 snRNA function (Figure 3C, left panel, Vector), but TRAP150 knockdown did not affect the expression of their PCPA transcripts (Supplementary Figure S3B). Nevertheless, TRAP150 overexpression induced CSPP of four genes (*CMTM8, GPR126, GSK3B* and *PLCH1*) (Figure 3C, lanes 4, 6, 8 and 14); intronic cryptic sites activated by TRAP150 were determined (Figure 3C, right panel). Therefore, our data suggested that TRAP150 has the potential to promote the splicing of PCPA transcripts.

### TRAP150 promotes cryptic 3′ splice site activation in PCPA transcripts

**Figure 4.**
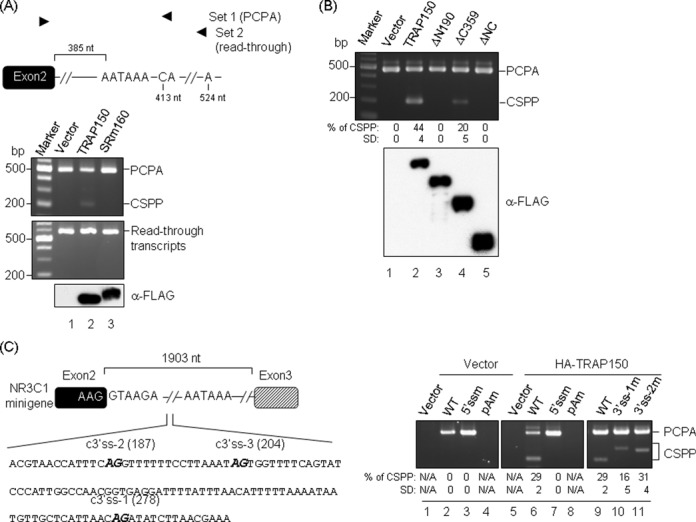
TRAP150 forces cryptic 3′ spice site activation in PCPA transcripts. (**A**) The empty or FLAG-tagged TRAP150 or SRm160 expression vector was transiently transfected in HEK293 cells. PCPA and CSPP of *NR3C1* were detected by 3′ RACE using primer sets as illustrated (top). Amplicons of primer set 1 and set 2 represent the PCPA/CSPP and read-through transcripts, respectively. Immunoblotting was performed using anti-FLAG (bottom). (**B**) The experiment was performed as in panel A, except that full-length or truncated TRAP150 was transiently expressed, and only primer set 1 was used for 3′ RACE. (**C**) The diagram (left) shows the *NR3C1* minigene and positions of the 5′ splice site and polyadenylation signal mutation. The cryptic 3′ splice sites (c3′ss) and their positions relative to the 5′ splice site are indicated. The pCEP4 empty vector or HA-TRAP150 expression vector was co-transfecteand with the wild-type (WT) or a mutant *NR3C1* minigene (5′ssm, pAm, 3′ss-1m or 3′ss-2m) into HeLa cells. 3′ RACE was performed as in panel B but using a vector-specific primer for the first round of PCR amplification (right). The splicing efficiency (% of CSPP) in panels B and C was calculated by relative CSPP expression levels (CSPP/CSPP+PCPA) from three independent experiments.

To assess the specificity of TRAP150 in the splicing of PCPA transcripts, we compared TRAP150 with another splicing factor, SRm160, which can enhance cleavage and polyadenylation in terminal exons by bridging U2 snRNP and CPSF (26). We overexpressed SRm160 in HEK293 cells and evaluated the expression and splicing of the *NR3C1* PCPA transcripts. Unlike TRAP150, overexpression of SRm160 did not elicit *NR3C1* CSPP expression (Figure 4A). Moreover, we found that TRAP150-activated splicing could not be detected in the read-through transcripts (Figure 4A), further indicating that CSPP resulted from a 3′ end processing-coupled splicing event. Next, we mapped the TRAP150 domains required for CSPP induction. After overexpressing truncated TRAP150 proteins, we observed that only ΔC359, but not ΔN190 or ΔNC, could generate CSPP of *NR3C1* (Figure 4B, lanes 3–5), consistent with our previous report that TRAP150-activated splicing requires its N-terminal domain (8).

To learn more about the molecular mechanism of TRAP150-induced composite terminal exon splicing, we used a minigene reporter of *NR3C1*, which spans exon 2 to exon 3 with an internally truncated intron 2 (Figure 4C, diagram). Overexpression of TRAP150 activated CSPP in the *NR3C1* minigene (Figure 4C, lane 6), as observed in the endogenous *NR3C1*. Next, we generated *cis*-element mutants of the *NR3C1* reporter. Regardless of TRAP150 overexpression, a mutation of the poly(A) signal disrupted the expression of both PCPA and CSPP (Figure 4C, lanes 4, 8), whereas the 5′ splice site mutation slightly promoted PCPA as expected (14) but impaired CSPP expression (lanes 3, 7). Mutation of the cryptic 3′ splice site (site 1) that was used in the above-detected CSPP activated an upstream 3′ splice site (site 2), resulting a longer CSPP product (lane 10, 3′ ss-1m). Furthermore, disruption of both sites 1 and 2 generated another CSPP product by using site 3 (lane 11; 3′ss-2m), indicating that TRAP150 could enforce suboptimal 3′ splice site utilization. Together, our result indicated that TRAP150 has a specific and strong effect in activating the splicing of PCPA transcripts.

### TRAP150-induced composite terminal exon splicing is enhanced under U1-deficient or transcription-compromised conditions

**Figure 5.**
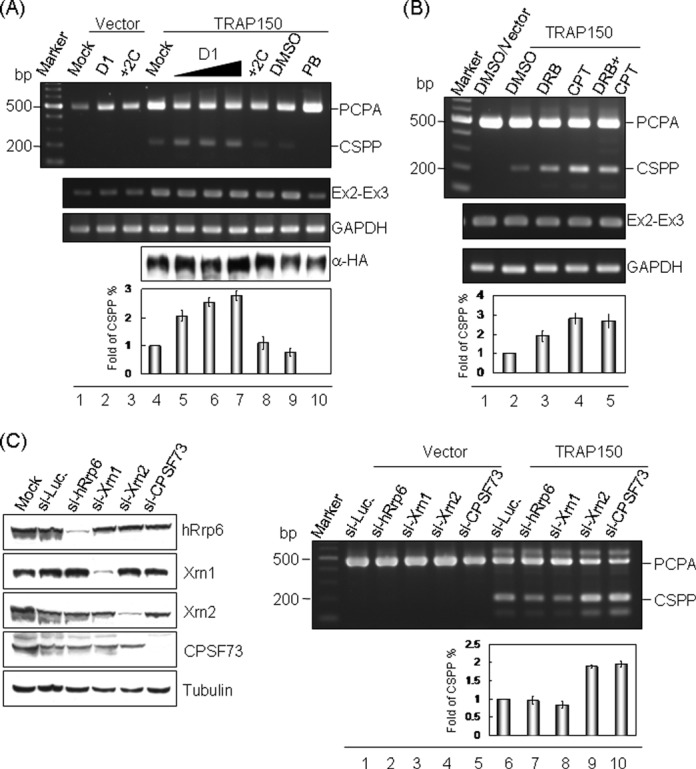
TRAP150-induced PCPA splicing is affected by functional U1 abundance and the transcription status. (**A**) HeLa cells were transfected with the following vectors: the empty (Vector) or HA-TRAP150 expression vector, the *NR3C1* minigene and the wild-type (D1) or mutant (+2C) U1 decoy expression vector. Lanes 5–7: an increasing amount of the D1 plasmid. At 24 h post-transfection, cells were treated with DMSO (vehicle, lane 9) or 1 μM PB (PB, lane 10) for 4 h. 3′ RACE (upper) and immunoblotting using anti-HA were performed. Ex2–Ex3 and GAPDH were examined as in Figure 3A. (**B**) As in panel A, transfectants were treated with 100 μM DRB (lane 3), 10 μM CPT (lane 4) or both (lane 5) for another 4 h. (**C**) HeLa cells were transfected with the indicated siRNAs for 48 h followed by transfection with the empty or HA-TRAP150 expression vector and the *NR3C1* minigene for another 24 h. Immunoblotting using specific antibodies as indicated (left) indicates the effectiveness of gene silencing by siRNA. In all three panels, the PCPA/CSPP transcripts of *NR3C1* were detected using 3′ RACE as in Figure 4C. In all panels, the bar graph shows the fold change of relative CSPP expression levels (CSPP/CSPP+PCPA); the percentage of CSPP expression of each lane was normalized to the negative control (lane 4, 2 and 6 in panels A, B and C, respectively). Average and standard deviation were obtained from three independent experiments.

Because debilitation of U1 snRNP enhances PCPA (14), we examined whether it has any effect on TRAP150-induced composite terminal exon splicing. We exploited a U1 decoy (27) to suppress the function of U1 snRNP. The U1 decoy D1 slightly increased the level of PCPA transcripts, whereas its mutant (+2C) had a minor effect (Figure 5A, lanes 2, 3), as reported (28). More importantly, we observed that the expression of CSPP was gradually enhanced with increasing amounts of the wild-type U1 decoy but was not affected by the mutant +2C (Figure 5A, lanes 4–8). CSPP expression was completely blocked by the splicing inhibitor PB (29), as expected (lane 10). This result indicated that limiting U1 snRNP facilitates PCPA and consequently promotes TRAP150-induced splicing in composite terminal exons.

Because TRAP150 associated with RNA pol II (Figure 2B), we examined whether the transcriptional status affects TRAP150-mediated splicing regulation of PCPA transcripts. We treated cells with the transcription elongation inhibitor DRB or camptothecin (30) and then examined PCPA/CSPP expression from the *NR3C1* minigene reporter. The result showed that DRB or camptothecin treatment enhanced TRAP150-induced CSPP by 2- to 3-fold (Figure 5B, lanes 3–5). This result suggested that PCPA splicing is a co-transcriptional event and that a slowing of transcriptional elongation may facilitate recruitment of splicing factors (reviewed in 31) and hence promotes TRAP150-induced splicing.

Finally, because termination of RNA polymerase II transcription is tightly coupled to the 3′ end processing of primary transcripts (reviewed in 32) and because several TRAP150-interacting factors, such as CPSF73 and the 5′-3′ exoribonuclease Xrn2 (Figure 1 and Supplementary Figure S5), have been implicated in such a coupling event, we assessed whether these factors may modulate the effect of TRAP150-induced PCPA splicing. Depletion of CPSF73 or Xrn2 using siRNA did not significantly affect PCPA but enhanced TRAP150-dependent CSPP expression by ∼2-fold (Figure 5C, right panel, lanes 4, 5, 9, 10). We also examined the effect of two other TRAP150-interacting factors, the exosome component Rrp6 and the 5′-3′ exoribonuclease Xrn1 (Supplementary Figure S5), but knockdown of either of these factors did not significantly affect PCPA splicing (Figure 5C, lanes 7, 8). We interpreted our result to mean that impaired transcription termination might retain prematurely terminated PCPA transcripts at sites of transcription (33) and allow splicing to occur.

### TRAP150 activates 3′ splice sites in composite but not in authentic terminal exons

**Figure 6.**
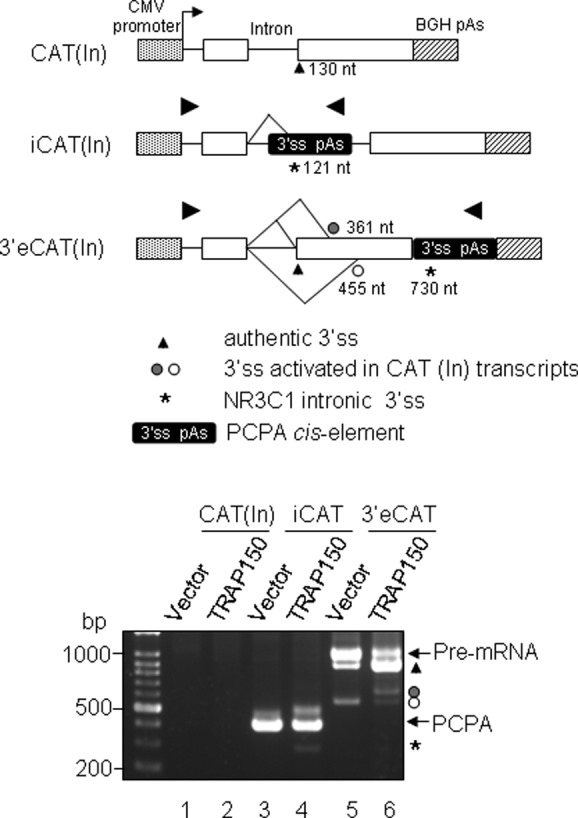
TRAP150 activates splicing in composite but not in authentic terminal exons. The diagrams (top) show the pcDNA-CAT(In) splicing reporters without or with the *NR3C1* fragment insertion into the intron or the 3′ exon, i.e. iCAT(In) and 3′eCAT(In), respectively. The asterisk depicts the cryptic 3′ splice site of NR3C1 intron 2. The empty or HA-TRAP150 expression vector was co-transfected with a splicing reporter into HeLa cells. PCPA products derived from the reporter were detected by 3′ RACE using the forward vector-specific primer and the reverse PCPA primer (bottom). Indicated products include pre-mRNA, PCPA, CSPP (asterisk) and mRNA transcripts using the authentic (triangle) or cryptic 3′ splice sites (open and close circles) in the 3′ exon of the parental CAT(In) reporter.

Next, we examined whether a minimal intronic fragment of *NR3C1* encompassing the poly(A) signal and the major cryptic 3′ splice site could function in heterologous genes. We inserted an *NR3C1* fragment spanning nucleotides −156 to +51 relative to the poly(A) signal (hereafter called PCPA *cis-*element) into the intron (derived from the human *β-globin* gene) of a previously used splicing reporter (20), resulting in pcDNA-iCAT(In) (Figure 6, diagram). This reporter exhibited enhanced splicing efficiency in HeLa cells (Supplementary Figure S6). However, under this condition, PCPA transcripts were still detectable (Figure 6, lane 3), indicating that intronic polyadenylation dominates over splicing in a certain fraction of the reporter transcript. Overexpression of TRAP150 induced CSPP (Figure 6, lane 4), as observed in the endogenous and minigene transcripts of *NR3C1* (Figures 3 and 5). Therefore, the PCPA *cis-*element could function in a heterologous intron and was still responsive to TRAP150.

To further examine whether the poly(A) signal dictates TRAP150-induced cryptic 3′ splice site usage in the authentic terminal exon, we placed the PCPA *cis-*element within the 3′ exon of the reporter (Figure 6, diagram; pcDNA-3′eCAT(In)). This reporter generated the authentic spliced product (Figure 6, lane 5, triangle) and activated a cryptic 3′ splice site downstream (open circle) of the authentic 3′ splice site but not the 3′ splice site of the PCPA *cis-*element (asterisk). Overexpression of TRAP150 dramatically enhanced the use of the authentic 3′ splice site (Figure 6, lane 6, triangle), although two minor cryptic products were still generated (open and filled circles). However, TRAP150 no longer activated the 3′ splice site(s) within the PCPA *cis-*element (lane 6). Together, our results indicated that PCPA is not functional in the exon context and that TRAP150 is unable to activate 3′ cryptic sites downstream of the authentic/strong one, and its splicing activity is likely independent of the polyadenylation signal.

## DISCUSSION

**Figure 7.**
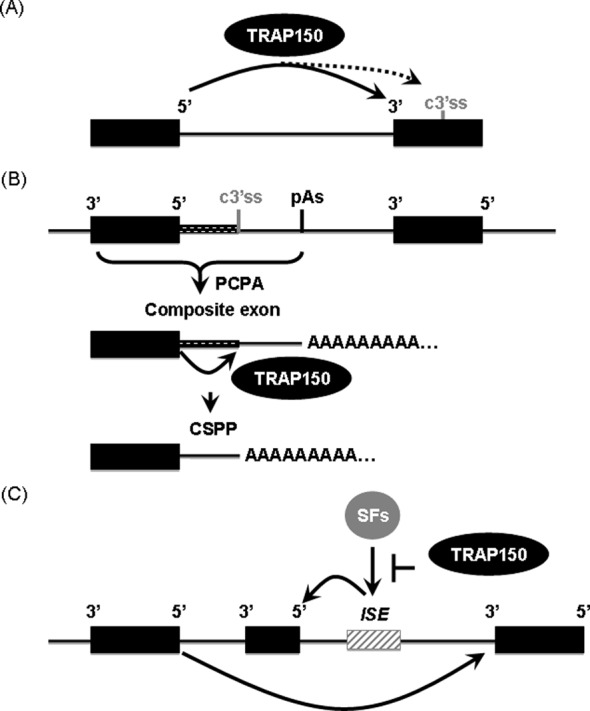
A model for TRAP150-activated splicing. (**A**) TRAP150 is essential for pre-mRNA splicing, as determined by a reporter assay (8). TRAP150 promotes the splicing efficiency of authentic introns (solid arrow line; 8), perhaps via a cross-intron bridging action, but it has less or no activity in activating cryptic 3′ splice sites in downstream exons (dashed arrow line) (this study). (**B**) TRAP150 activates cryptic splicing of prematurely terminated transcripts (composite terminal exon) that are produced by promoter-proximal cleavage/polyadenylation (solid arrow line) (this study). (**C**) TRAP150 represses upstream exon inclusion by sequestering or suppressing splicing activators (SFs) that bind to the intronic enhancers (10 and Supplementary Figure S1C in this study).

We previously reported that TRAP150 co-transcriptionally promotes pre-mRNA splicing (8). The present study shows that TRAP150 has a specific role in promoting cryptic splicing of PCPA transcripts that are generated from genes containing composite exons (Figure 7).

### TRAP150 activates the splicing of PCPA transcripts

We show that TRAP150 co-fractionated and is associated with RNA pol II and interacts specifically with U1 snRNP and the CPSF complex (Figures 1 and 2). We have previously shown that the N-terminal RS domain of TRAP150 is essential for splicing activation (8). This domain may also participate in intermolecular RS domain interactions with those identified factors, which possibly involves (Figure 2). The interaction of TRAP150 with RNA pol II supported the co-transcriptional splicing enhancement activity of TRAP150 (8). Moreover, this finding provides a hint that TRAP150 functions with either of its interacting RNA processing complexes or even connects the transcription, splicing and polyadenylation during pre-mRNA processing. It has been reported that the interaction between splicing and cleavage/polyadenylation factors may facilitate and ensure proper 3′ end processing (34,35). For example, elevated levels of SRm160 stimulate cleavage followed by polyadenylation (26). In our study, we did not rigorously evaluate the role of TRAP150 in 3′ processing *per se*, but our data indicate that TRAP150 could neither modulate terminal/3′-most alternative polyadenylation selection nor facilitate intronic polyadenylation (Figure 3 and Supplementary Figure S2). The effect of TRAP150 on CT/CGRP terminal exon selection is exceptional and perhaps depends on the presence of a pseudoexon enhancer (Supplementary Figure S1 and Figure 7C).

PCPA represents a special case of alternative polyadenylation that frequently occurs near the 5′ end of genes and is accompanied by premature termination. PCPA converts a poly(A) site-bearing intron into a composite terminal exon. Although TRAP150 knockdown had no significant effect on the PCPA process, its overexpression activated the splicing of PCPA transcripts (Figure 3). This effect was not observed for SRm160, which interacts with CPSF and promotes the activity of cleavage factors (26). Therefore, TRAP150 and SRm160 may function differently, although both interact with CPSF. Moreover, our data also support that TRAP150 and BCLAF1 play distinct roles in post-transcriptional regulation of mRNA biogenesis, as previously observed (9). Furthermore, the observation that TRAP150 could interact with RNA pol II bearing phosphorylated serine-5 residues in the C-terminal repeated motif might support the function of TRAP150 in composite terminal exon splicing that occurs in the 5′ end region of the transcripts (Supplementary Figure S5). Nevertheless, we cannot completely exclude the possibility that TRAP150 has a role in 3′ end processing, such as facilitating CPSF loading to (specific) polyadenylation sites.

### Possible role for TRAP150 in composite terminal exon splicing

Our data reveal that TRAP150 overexpression activates the authentic 5′ and cryptic 3′ splice sites in PCPA. We gathered that the CPSF complex, which binds to the intronic poly(A) site, might dictate TRAP150 in 3′ splice site selection in composite exons so that the activated 3′ splice sites are close to (≤100 nt upstream) the cleavage/polyadenylation site (Figures 3 and 7). We note that, when CSPP was generated upon TRAP150 overexpression, the level of PCPA transcripts was reduced, which suggests that TRAP150-activated PCPA splicing occurs following cleavage/polyadenylation. This possibility is supported by the failure to detect TRAP150-activated intronic splicing in the read-through transcripts (Figure 4). Moreover, TRAP150 has strong activity in promoting splicing of authentic but not the cryptic 3′ splice site(s) in the downstream exon (Figures 6 and 7); such an activity of TRAP150 is likely independent of CPSF.

Treatment of cells with DRB or camptothecin or knockdown of Xrn2 or CPSF73 enhanced CSPP expression (Figure 5). Perhaps, under compromised transcriptional conditions, the growing pre-mRNA has more opportunities to interact with TRAP150 and the splicing machinery, and thereby PCPA splicing is enhanced. This result also indicates that transcription and TRAP150-induced composite terminal exon splicing are kinetically coupled. Composite exon splicing was inhibited by the splicing inhibitor PB, as expected (Figure 5). A U1 decoy, when expressed at levels that had no significant deleterious effect on pre-mRNA splicing, enhanced TRAP150-activated composite terminal exon splicing (Figure 5). This was probably a consequence of enhanced expression of PCPA transcripts during partial blockage of U1 snRNP (14,19,28).

### Functional implication of TRAP150-induced composite terminal exon splicing

The biased distribution of the 5′ splice site-poly(A) site axis toward the sense strand of genes ensures the expression of long/functional transcripts (17,18,36). Nevertheless, cryptic polyadenylation signals are widely distributed throughout genes. U1 snRNP suppresses inappropriate poly(A) signal usage at sites including those in introns, which represents a type of RNA surveillance (14,18). However, PCPA transcripts are still detectable when U1 is fully functional (37; our observation), although it is enhanced in cancer cells or transiently activated cells owing to transient U1 shortage (19). The question of whether TRAP150-induced composite terminal exon splicing is merely a futile activity or has any cellular effects warrants future experiments. Nevertheless, PCPA has been implicated in regulating signal transduction and even in a cascade of regulated alternative polyadenylation events (28,38). Therefore, one may suspect that spliced PCPA transcripts also have physiological roles. Overexpression of TRAP150 promotes composite terminal exon splicing, which might increase the diversity of the PCPA transcripts or even affect PCPA-regulated cellular function(s). On the other hand, we previously reported that TRAP150, when tethered to a pre-mRNA, downregulates its mRNA expression (8). However, the notion that PCPA transcripts and their spliced products are polyadenylated argues against their instability. Future studies will aim to determine the biochemical and biological consequence of TRAP150-induced composite terminal exon splicing.

## SUPPLEMENTARY DATA

Supplementary Data are available at NAR Online.

SUPPLEMENTARY DATA
