# Supplementary material for: TRAP150 activates splicing in composite terminal exons
Source: Nucleic Acids Res. 2014 Oct 17;42(20):12822–32. doi: 10.1093/nar/gku963 (PMC4227790; doi:10.1093/nar/gku963)
Supplement: SUPPLEMENTARY DATA [file supp_42_20_12822__index.html]

TRAP150 activates splicing in composite terminal exons — TRAP150 activates splicing in composite terminal exons — SUPPLEMENTARY DATA 

# TRAP150 activates splicing in composite terminal exons

## SUPPLEMENTARY DATA

**Files in this Data Supplement:**

- SUPPLEMENTARY DATA
