# Supplementary material for: TRAP150 activates splicing in composite terminal exons
Source: Nucleic Acids Res. 2014 Oct 17;42(20):12822–32. doi: 10.1093/nar/gku963 (PMC4227790; doi:10.1093/nar/gku963)

Supplementary materials and methods

Plasmids and antibody

The splicing reporters used in this study included pCEP4-E1a (1), pTS23D containing rat α-Tropomyosin exons 1-4 (Ref. 2; a gift of C. W. J. Smith, University of Cambridge) and pCTG6 containing the first exon of the adenovirus major late gene fused to CT/CGRP exons 4-6 (Ref. 3; a gift of H. Lou, Case Western Reserve University). The human BCLAF1 cDNA was PCR amplified and in-frame inserted into the pcDNA3.1 and pCEP4 vector to generate FLAG-tagged and HA-tagged BCLAF1 expression vector, respectively.

Anti-Ser5 phosphorylated CTD (ab5131) antibody was from Abcam.

***In vivo* splicing and reverse transcription-PCR (RT-PCR)**

For *in vivo* splicing assay, 0.5 µg of the reporter plasmid was co-transfected with 2.5 µg of the effecter plasmid into ~1×10^6 HeLa cells. For the knockdown experiments, cells were transfected with 100 nM siRNAs for 48 hr prior to reporter transfection. At 24 h post-transfection, RNAs were recovered for reverse transcription-PCR analysis using specific primers (Supplementary Table S1). The RT-PCR products of α-Tropomyosin were restriction digested with XhoI or PvuII to differentiate mutually exclusive usage of exons 2 and 3.

All other materials and methods are described in the main text.

SUPPLEMENTARY DATA

Supplementary Figure S1. TRAP150 suppresses the exon inclusion mediated by the intronic splicing enhancer but has no apparent effect on alternative 5' splice site or 5' exon selection. The schemes (top of each panel) denote the splicing reporters and the primers or the restriction enzymes used to detect the spliced products. Expression plasmid encoding the full-length (WT) or truncated HA-tagged TRAP150 or BCLAF1 was each co-transfected with the E1A reporter (A) or *α-tropomyosin* (α-TM, B) reporter or pCTG6 reporter (C) into HeLa cells for 24 h. For the knockdown experiments, cells were transfected with indicated siRNAs for 48 hr prior to the transfection of the reporter plasmids. Twenty-four hr later, total RNA from the transfectants was prepared for RT-PCR analysis. The splicing efficiency of pCTG6 was evaluated by the ratio of CT exon 4 inclusion (Ex4+/total) from three independent experiments. P: PvuII digestion; X: XhoI digestion. RSV: Rous sarcoma virus promoter. ADE: adenovirus major late gene. pY/5'ss: the pseudo exon composed of the polypyrimidine tracts immediately followed by the 5' splice site. Overexpressed proteins were detected by immunoblotting using α-HA. Knockdown of TRAP150 and BCLAF1 were examined by immunoblotting using α-TRAP150 and α-BCLAF1, respectively. Tubulin served as the loading control (bottom).

Supplementary Figure S2. TRAP150 may not be involved in general alternative polyadenylation of terminal exons. (A) The schemes illustrate the genes examined in this study, which undergo alternative cleavage/polyadenylation in the terminal exons. Two distinct reverse primers were used to distinguish the transcripts using the proximal (P) or distal (D) polyadenylation site as described (4,5). (B) siRNA transfection and RT were carried out as described in Figure 3A, lanes 1-4. Quantitative PCR was performed to examine the relative level of the short (P) or long (D) form transcripts. The bar graph shows the relative usage of the two polyadenylation sites (P versus D); the result was obtained from three independent experiments. Immunoblotting using anti-TRAP150 was shown in the bottom.

Supplementary Figure S3. Knockdown of TRAP150 does not affect the expression level of PCPA transcripts. (A) The diagrams show the candidate genes bearing the intronic poly(A) signal (pAs). The size of the intron (in nt) and the position of the pAs are denoted. (B) siRNA transfection and RT were performed as described in Figure 3A, and the PCPA transcripts of each candidate gene were analyzed using 3' RACE as described in Figure 3.

Supplementary Figure S4. BCLAF1 does not activate the expression of the *NR3C1* CSPP transcript. The effect of BCLAF1 overexpression was examined on the PCPA processing of endogenous (A) or *NR3C1* minigene (B). Transfection, RT-PCR and 3'RACE were performed essentially as described in Figure 3. To exclude the possibility that no CSPP indication by BCLAF1 was due to its poor expression, the *in vivo* splicing assay was performed using the pCTG6 reporter (panel A, middle) as described in Supplementary Figure S1. BCLAF1 overexpression reduced CT exon 4 utilization. Immunoblotting using the indicated antibodies was shown below.

Supplementary Figure S5. TRAP150 interacts with Xrn1, Xrn2, hRrp6, and phospho-Ser 5-containing RNA pol II. Transfection, immunoprecipitation and

RNase treatment were performed essentially as described in Figure 1. Immunoblotting was conducted using the indicated antibodies.

Supplementary Figure S6. Insertion of the *NR3C1* intron 2 fragment enhances the splicing activity of the CAT(In) reporter. The diagrams (top) show different CAT(In)-derived reporters as described in Figure 6; the primers used for RT-PCR are denoted. Transfection of HA-tagged TRAP150 and RT-PCR (bottom) was performed as described (6). The lower panel shows immunoblotting using anti-HA.

Supplementary References

1. Lai, M.C., Kuo, H.W., Chang, W.C. and Tarn, W.Y. (2003) A novel splicing regulator shares a nuclear import pathway with SR proteins. *EMBO J*, **22**, 1359-1369.
2. Gromak, N., Rideau, A., Southby, J., Scadden, A.D., Gooding, C., Huttelmaier, S., Singer, R.H. and Smith, C.W. (2003) The PTB interacting protein raver1 regulates alpha-tropomyosin alternative splicing. *EMBO J*, **22**, 6356-6364.
3. Lou, H., Helfman, D.M., Gagel, R.F. and Berget, S.M. (1999) Polypyrimidine tract-binding protein positively regulates inclusion of an alternative 3'-terminal exon. *Mol Cell Biol*, **19**, 78-85.
4. Tian, B., Pan, Z. and Lee, J.Y. (2007) Widespread mRNA polyadenylation events in introns indicate dynamic interplay between polyadenylation and splicing. *Genome Res*, **17**, 156-165.
5. Berg, M.G., Singh, L.N., Younis, I., Liu, Q., Pinto, A.M., Kaida, D., Zhang, Z., Cho, S., Sherrill-Mix, S., Wan, L. *et al.* (2012) U1 snRNP determines mRNA length and regulates isoform expression. *Cell*, **150**, 53-64.
6. Lee, K.M., Hsu Ia, W. and Tarn, W.Y. (2010) TRAP150 activates pre-mRNA splicing and promotes nuclear mRNA degradation. *Nucleic Acids Res*, **38**, 3340-3350.

Supplemental Table S1. The list of the primers used in this study

Primer	Sequence
oligo dT18-XbaKpnBam	5’ CTG ATC TAG AGG TAC CGG ATC CTT TTT TTT TTT TTT TTT T-3’
XbaKpnBam	5’-CTG ATC TAG AGG TAC CGG ATC C-3’
pcDNA-5’	5’ GTT AAG CTT GGT ACC GAG CT-3’
NR3C1Ex2-1	5’-GCC AAG GAT CTG GAG ATG AC-3’
NR3C1Ex2-2	5’-CTC TGA ACT TCC CTG GTC GAA C-3’
NR3C1 Ex3R	5’-GGC GGC CTC GAG CTT CCA CTG CTC TTT TGA AGA AAA C-3’
NR3C1PCPAR	5’-TTT TTT TTT TTT GAC TAT GTA AGT-3’
NR3C1524R	5’-TAA CAG AAG ATT CAG TTT GAA GAA TAG-3’
CDC42F	5’-CTG AAG GCT GTC AAG TAT GTG G-3’
CDC42R1 (P)	5’-TCT TTC TAC AGT AGT GGG ACA GGA-3’
CDC42R2 (D)	5’-CTG TGC AGA AAG GGC TCT G-3’
GABPB1F	5’-GGT CAG CCC ATC ATT GTG AC-3’
GABPB1R1 (P)	5’-CAA TAT TTA TTT ATT GAG GGC TTG C-3’
GABPB1R2 (D)	5’-GCT TCA TCC AGC TGT TTC TG-3’
UBAP2LF	5’-TGG TGT GAA TGT CAG TGT GAA TGC-3’
UBAP2LR1 (P)	5’-ACA AAA CAC AGC CCC CAG C-3’
UBAP2LR2 (D)	5’-GCA AGT TGA AGG AAG CAG CAG G-3’
STK17Aex2-1	5’-GAA CTA GCA CAA GAC AAT CCT TGG-3’
STK17APCPAR	5’-TTT TTT TTT TTT TTT AAG GAT TAT AAA ATG CCT-3’
SEMA3Cex1-1	5’-GAC TGG AGA CAC AAG CGC ATC-3’
SEMA3Cex1-2	5’-GCT GAT TTC TTT CAA CCT CG-3’
SEMA3CPCPAR	5’-TTT TTT TTT TTT TTT GTC CAG TAA AGA AAT TTA T-3’
ACN9ex1-1	5'-CTC AAA TCC CTG GGC GAC-3'
ACN9ex1-2	5'-GTA CGT GAA AGA CGA ATT TAG GAG AC-3'
ACN9PCPAR	5'-TTT TTT TTT TTT GAA AAC TAA TAA GAG AAC-3'
CMTM8ex1-1	5'-TTC GCA GAG AAC TTC TCC ACC AGC-3'
CMTM8ex1-2	5'-CGG CTT CCT CAT CGT GGC CGA G-3'
CMTM8PCPAR	5'-TTT TTT TTT TTT GAT CTT AGG AAG C-3'
GPR126ex2-1	5'-GAT GTT TCG CTC AGA TCG AAT G-3'
GPR126ex2-2	5'-CTG CCA TTG GAA ATG GAA GC-3'
GPR126PCPAR	5'-TTT TTT TTT TTT GTA TAT TTA TTC AAC AGT AG-3'
GSK3Bex1-1	5'-CGC GAA GAG AGT GAT CAT GTC AG-3'
GSK3Bex1-2	5'-GCC GGT GCA GCA GCC TTC AGC-3'
GSK3BPCPAR	5'-TTT TTT TTT TTT GTC CTT CTC TTT ATT C-3'
NF1Aex2-1	5'-GCT CTA ATC CAG GGC TCT GTG TC-3'
NF1Aex2-2	5'-GCA TAC TTT GTG CAT GCA GCA G-3'
NF1APCPAR	5'-TTT TTT TTT TTT GTC AGT TAA AAT ATA CTC-3'
PLCE1ex1-1	5'-TTA CCT TGT TAA AGG ATG GAT GTG-3'
PLCE1ex1-2	5'-GCT GGA GGC TTA AAG AAG ACC AG-3'
PLCE1PCPAR	5'-TTT TTT TTT TTT GCA AAA AGA AGA GCA-3'
PLCH1ex1-1	5'-CTG CTC TTG CCA ATA ATG TGT AAT AG-3'
PLCH1ex1-2	5'-ATG GCA GAC CTT GAA GTG TAT AAA AAC-3'
PLCH1PCPAR	5'-TTT TTT TTT TTT TAG AAA GTT AAA ATA TAG C-3'

P598 (T7)	5'-TAA TAC GAC TCA CTA TAG GG-3'
CAT(In)F	5'-GGA GCT AAG GAT CCT AAA ATG GAG-3'
p861 (CAT(In)R)	5'-GTA TTC ACT CCA GAG CGA TG-3'
SV40 (P2/α-TMF)	5'-GCA AGC TTG AGT GCC AGC GAG TAG-3'
P1 (E1aR)	5'-GGT CTT GCA GGC TCC GGT TCT GGC-3'
TM4 (α-TMR)	5'-CAG AGA TGC TAC GTC AGC TTC AGC-3'
DS8 (RSV)	5'-TTG ACC ATT CAC CAC ATT GGT GTG C-3'
4967 (CT/CGRP)	5'-GAG TTT AGT TGG CAT TCT GG-3'
GN (CT/CGRP)	5'-CTG CTC AGG CTT GAA GGT CC-3'

Supplementary Table S2. Gene name abbreviations in this study

Abbreviation	Full gene name
ACN9	ACN9 homolog (S. cerevisiae)
BCLAF1 (Btf)	BCL2-associated transcription factor 1
CDC42	Cell division cycle 42
CMTM8	CKLF-like MARVEL transmembrane domain containing 8
GABPB1	GA binding protein transcription factor, beta subunit 1
GPR126	G protein-coupled receptor 126
GSK3B	Glycogen synthase kinase 3 beta
NF1A	Nuclear factor I/A
NR3C1	Nuclear receptor subfamily 3, group C, member 1
PLCE1	Phospholipase C, epsilon 1
PLCH1	Phospholipase C, eta 1
SEMA3C	Sema domain, immunoglobulin domain (Ig), short basic domain, secreted, (semaphorin) 3C
STK17A	Serine/threonine kinase 17a
TRAP150 (THRAP3)	Thyroid hormone receptor-associated protein of 150 kDa (Thyroid hormone receptor-associated protein 3)
UBAP2L	Ubiquitin associated protein 2-like 1

Supplementary Figure S1

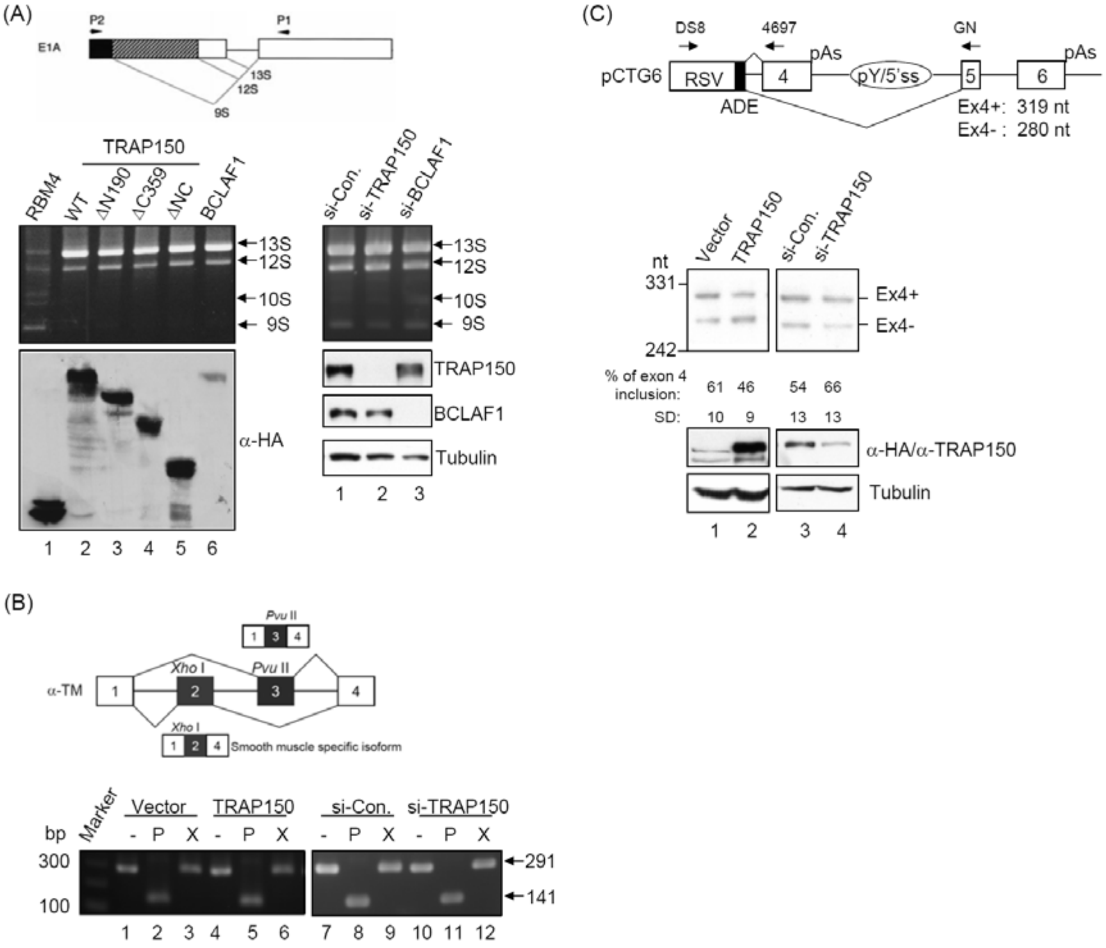

Supplementary Figure S2

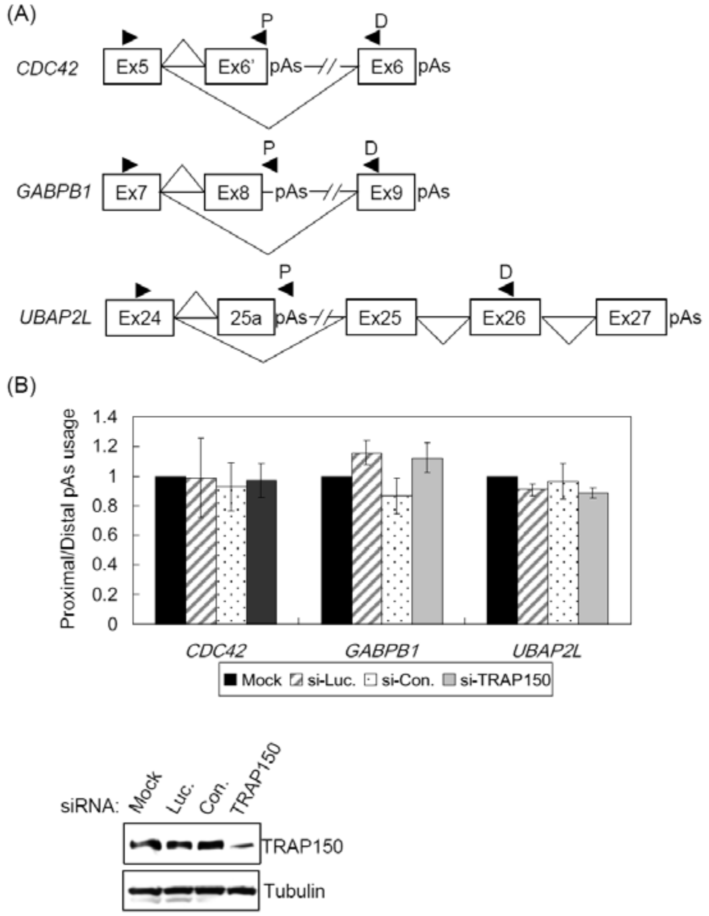

Supplementary Figure S3

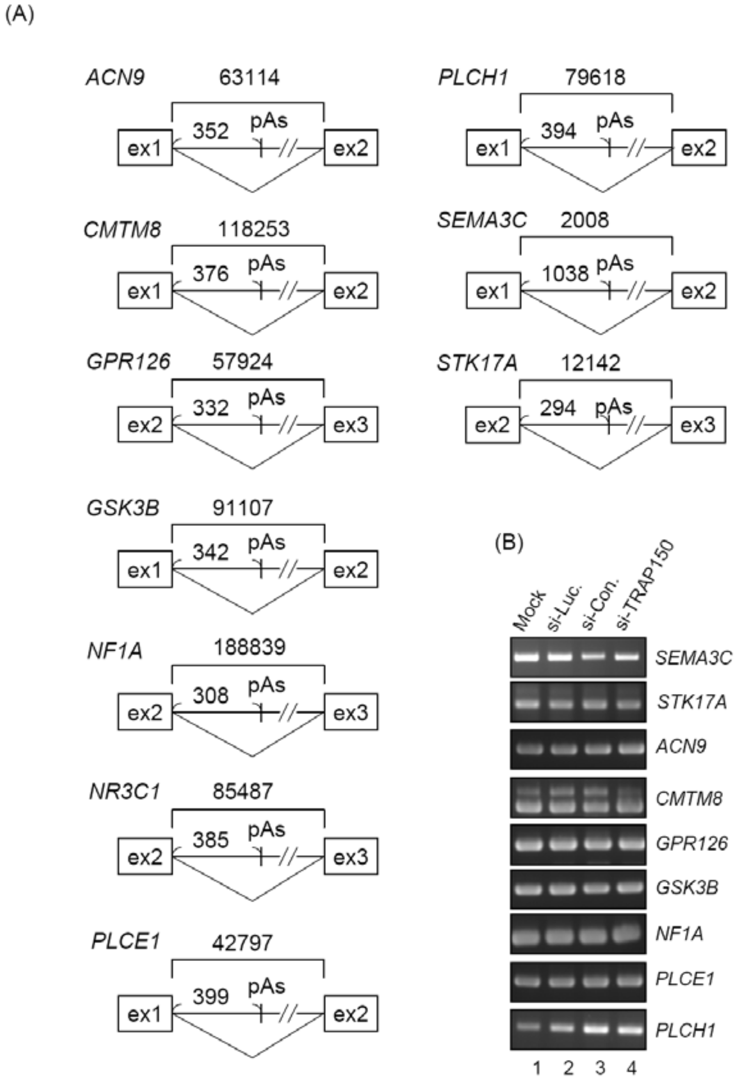

Supplementary Figure S4

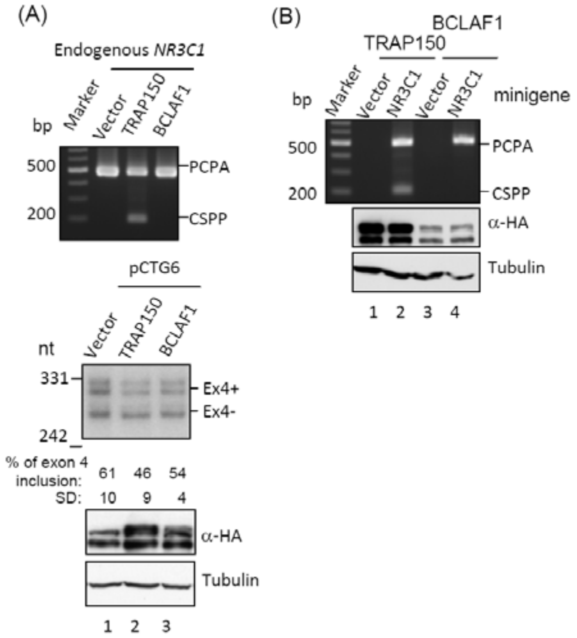

Supplementary Figure S5

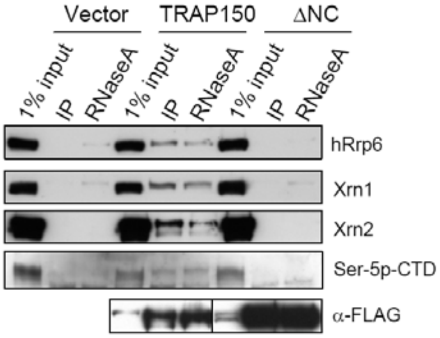

Supplementary Figure S6

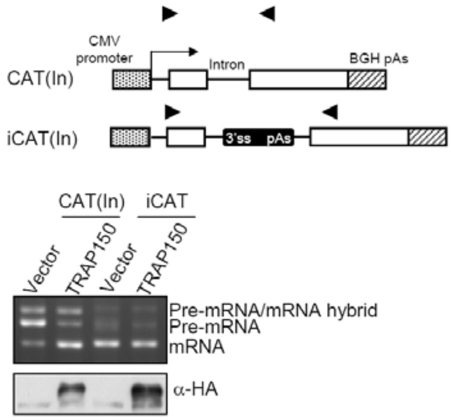

Supplement: SUPPLEMENTARY DATA [file supp_gku963_nar-01692-a-2014-File009.pdf]
